# The Joint Adaptive Kalman Filter (JAKF) for Vehicle Motion State Estimation

**DOI:** 10.3390/s16071103

**Published:** 2016-07-16

**Authors:** Siwei Gao, Yanheng Liu, Jian Wang, Weiwen Deng, Heekuck Oh

**Affiliations:** 1 College of Computer Science and Technology, Jilin University, Changchun 130012, China; gaosw14@mails.jlu.edu.cn (S.G.); yhliu@jlu.edu.cn (Y.L.); 2 Key Laboratory of Symbolic Computation and Knowledge Engineering of Ministry of Education, Jilin University, Changchun 130012, China; 3 State Key Laboratory of Automotive Simulation and Control, Jilin University, Changchun 130012, China; kdeng@jlu.edu.cn; 4 Department of Computer Science and Engineering, Hanyang University, Ansan 426791, Korea; hkoh@cse.hanyang.ac.kr

**Keywords:** Joint Kalman Filter, innovation-based adaptive estimation, motion state estimation, data fusion

## Abstract

This paper proposes a multi-sensory Joint Adaptive Kalman Filter (JAKF) through extending innovation-based adaptive estimation (IAE) to estimate the motion state of the moving vehicles ahead. JAKF views Lidar and Radar data as the source of the local filters, which aims to adaptively adjust the measurement noise variance-covariance (V-C) matrix ‘R’ and the system noise V-C matrix ‘Q’. Then, the global filter uses R to calculate the information allocation factor ‘β’ for data fusion. Finally, the global filter completes optimal data fusion and feeds back to the local filters to improve the measurement accuracy of the local filters. Extensive simulation and experimental results show that the JAKF has better adaptive ability and fault tolerance. JAKF enables one to bridge the gap of the accuracy difference of various sensors to improve the integral filtering effectivity. If any sensor breaks down, the filtered results of JAKF still can maintain a stable convergence rate. Moreover, the JAKF outperforms the conventional Kalman filter (CKF) and the innovation-based adaptive Kalman filter (IAKF) with respect to the accuracy of displacement, velocity, and acceleration, respectively.

## 1. Introduction

The motion state estimation of the forward-moving vehicle is a prerequisite for automatic driving vehicles, and its main outputs are the relative transverse longitudinal velocity and acceleration, and relative location. Currently, Lidar and Radar are commonly used as distance measurement sensors in automatic driving systems, which were already fully demonstrated by many teams at the DARPA Urban Challenge [1,2,3]. Lidar has high accuracy and wide measuring range, and can immediately obtain targets’ displacement, and thus can simply calculate velocity, acceleration, and other states. Laser measurements however easily suffer from attenuation over the air, and the perception accuracy happens to sharply decline due to the serious noise, and thus Lidar fails to work normally in bad weather [4,5,6]. Radar is another common distance measurement sensor that can easily obtain targets’ displacement, velocity, and acceleration. Despite a lower accuracy than Lidar, Radar is better at penetrating through smoke and dusts, and thus is robust against bad weather conditions. Hence, an obvious step is to fuse Lidar and Radar sensors in order to highlight their respective advantages and to compensate their mutual shortcomings. By exploiting the associated properties with the different frequency spectra, these sensors can become excellent candidates for data processing and fusion systems [7].

The Kalman filter is widely used to estimate the motion state of a dynamic target. However, the CKF [8] needs to ensure the measurement noise V-C matrix R and the system noise V-C matrix Q precisely enough in order to achieve the best filtering performance, but in fact, R and Q often sensitively fluctuate with the varying accuracy of sensors and data sampling frequency. When encountering bad weather and/or frequently changing environments, the CKF can even output a divergent filtered result.

To address the above issues, we propose a JAKF that employs Lidar and Radar to estimate the motion state of vehicles. It is a multi-layer filter through extending Federated Fusion-Reset (FR) Mode [9] that splits a complete filter into two local filters and one global filter. JAKF treats the Lidar and Radar sensors as the source of the Local Filter (LF) to concurrently work with IAE [10]. First, the data collected by the Lidar and Radar sensors are preprocessed by coordinate transformation and time synchronization. Then, the preprocessed data are input into two LFs from the Lidar and Radar sensors, respectively. Finally, the locally filtered results along with the corresponding system noise *Q*, measurement noise *R*, and filtered result V-C matrix *P* are delivered to the Global Filter (GF). During the process of joint filtering, the information allocation factor β needs to be calculated by R in LF. β can adjust the weight of the state vectors X and P of LF towards the optimal fusion result. When the performance of Lidar happens to worsen, its R will be increased accordingly, then β will be reduced, and thus the noise pollution posed by this sensor can be mitigated. The JAKF combines the features of Lidar and Radar, so it is a stable and well-adaptive method to estimate the motion state of the vehicle ahead.

In this paper, an adaptive decision should be made by balancing the performance between the Lidar and Radar. The main contributions can be summarized as follows: (i) we propose a precise and robust method for estimating vehicle motion state by extending the CKF; (ii) extensive simulations and experiments to compare the accuracy and stability of CKF, IAKF, and JAKF are conducted and (iii) we apply a multi-sensor system to vehicle motion state estimation and provide the pros and cons of Lidar and Radar.

The remainder of this paper is structured as follows: Section 2 overviews a number of motion state estimation methods in the recent literature; Section 3 introduces the proposed JAKF in details, and gives the coordinate transformation and time synchronization in particular; Section 4 presents our extensive simulation and experimental results to verify the accuracy and stability of the JAKF; Finally, Section 5 draws some conclusions and suggests the next work.

## 2. Related Work

The key to accurately estimate the motion state is to get useful information from a large number of sensor data. A good model of the target will undoubtedly facilitate this information extraction to a great extent [11]. Over the past three decades, many kinds of estimation models have been proposed, and the Kalman filter in different forms has been widely used in these models, e.g., extended Kalman filter, unscented Kalman filter, and other nonlinear filters based on the conventional Kalman filter. According to the number of the installed sensors, they can be divided into single-sensor filters and multi-sensor filters.

Single-sensor filters have high efficiency and usually use high precision sensors to improve the accuracy of results. There are many common vehicle-mounted sensors, e.g., INS, Lidar, radar, camera, accelerometer, and more others. Lee et al. [12] proposed an interactive multiple model (IMM) estimator based on fuzzy weighted input estimation for tracking a maneuvering target. The fuzzy logic theory is utilized to construct the fuzzy weighting factor to improve the input estimation method and that is used to compute the unknown acceleration input for the modified Singer acceleration model. The modified Singer acceleration model combined with the fuzzy weighted input estimation method can track the maneuvering target effectively. Hollinger et al. [13] proposed Ground vehicle-based LADAR for standoff detection of roadside hazards, and discussed detection of roadside hazards partially concealed by light to medium vegetation. Hong et al. [14] proposed a car test for the estimation of GPS/INS alignment errors, and presented car test results on the estimation of alignment errors in the integration of a low-grade inertial measurement unit (IMU) with accurate GPS measurement systems. An iterative scheme was used to improve the estimation of the alignment errors during post-processing. The scheme was shown to be useful when the test car did not have sufficient changes in motion due to limitations in its path. Xian et al. [15] proposed a robust innovation-based adaptive Kalman filter for INS/GPS land navigation. A robust IAE-AKF algorithm was presented in this paper, which evaluates the innovation sequence with Chi-squared test and revises the abnormal innovation vector. The new algorithm possesses high accuracy and stability, and also has the ability to prevent the filtering from diverging even in a rigorous GPS measurement environment.

In practice, the single-sensor filter generally fails to work as excellently as declared due to its performance limitations. If one filter can be calibrated by another one, the eventual filtered results can be more accurate. Multi-sensor filters aim to solve this issue through integrating the respective superiority of different sensors. Multi-sensor filters fuse the results of different sensors into an optimal filtered result at the cost of efficiency in order to improve fault tolerance and robustness. Han et al. [16] proposed maneuvering obstacle motion state estimation for intelligent vehicles using an adaptive Kalman filter based on the current statistical model, and developed such a system that uses a radar and GPS/INS. They introduced a current statistical (CS) model from the military field, which uses the modified Rayleigh distribution to describe acceleration. The adaptive Kalman filter based on CS model was used to estimate the motion state of the target. Mirzaei et al. [17] proposed a Kalman filter-based algorithm for IMU-Camera systems. The proposed method does not require any special hardware (such as a spin table or 3-D laser scanner) other than a calibration target. Furthermore, they employed the observability rank criterion based on Lie derivatives and proved that the nonlinear system describing the IMU-camera calibration process is observable. Sarunic et al. [18] proposed hierarchical model predictive control of UAVs performing multitarget-multisensor tracking, which enables implementation of a computationally feasible and suboptimal solution that takes into account both short-term and long-term goals. Hostettler et al. [19] proposed joint vehicle trajectory and model parameter estimation using roadside sensors, and introduced how a particle smoother-based system identification method can be applied for estimating the trajectory of road vehicles. As for sensors, they adopted a combination of an accelerometer measuring the road surface vibrations and a magnetometer measuring magnetic disturbances mounted on the side of the road.

There are some discussions about making structure of multi-sensor filter more stable. Naets et al. [20] proposed an online coupled state/input/parameter estimation approach for structural dynamics, which uses a parametric model reduction parameter technique. The reduced model is coupled to an Extended Kalman Filter (EKF) with augmented states for the unknown inputs and parameters. This leads to an efficient framework for estimation in structural dynamics problems. Chatzi et al. [21] compared the unscented Kalman filter and particle filter methods for nonlinear structural system identification with non-collocated heterogeneous sensing. The use of heterogeneous, non-collocated measurements for non-linear structural system identification is explored. They also proposed online correction of drift in structural identification using artificial white noise observations and an unscented Kalman filter [22]. The method relies on the introduction of artificial white noise (WN) observations into the filter equations, which is shown to achieve an online correction of the drift issue, thus yielding highly accurate motion data. The above literature focuses on how to fuse the data from the same type of multiple sensors to improve the accuracy, and the final accuracy is higher than that of any other single sensor. In this paper, the data collected by different types of multiple sensors, i.e Lidar and Radar, are fused by the FR mode. Both the accuracy and system stability are improved together. When any one type of equipped sensors is on outage, the other can still optimize the result by feedback. In addition, the proposed method synchronizes the different rates of various sensors by Lagrange three-point interpolation to realize the multi-rate Kalman filter.

In a word, the current main efforts are made to improving the accuracy of the filtered results. However, when suffering from continuous high noise, none of the related sensors would keep working as expected. Any sensor losing effectivity undoubtedly decreases the overall performance, so an adaptive control decision should be made by a tradeoff between accuracy and stability. Considering the two factors, the proposed JAKF employs the high-accuracy Lidar and Radar as the source of input data, and synthesizes the respective advantages of IAE and FR to realize the multi-sensor adaptation. In addition, Chi-square hypothesis test and correction decreases measurement error, and coordinate transformation and time synchronization decreases fusion error. Therefore, JAKF is more accurate and stable than the conventional federated Kalman filter and single-sensor adaptive Kalman filter using Lidar or Radar.

## 3. Method

This section first briefly introduces the structure of JAKF, and then explains the local and global filter in details, and at last provides two post-processing operations: coordinate transformation and time synchronization.

### 3.1. The Structure of JAKF

**Figure 1:**
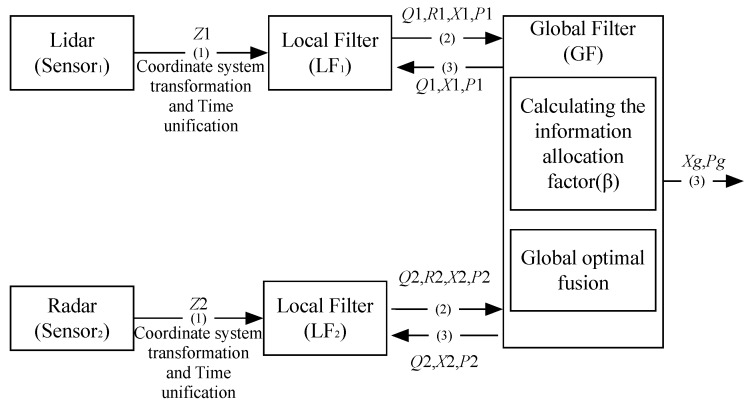
The structure and working process of JAKF.

JAKF is a two-step processing method for partition estimation. Figure 1 shows the structure and working process of JAKF. (1) Sensors send the current collected data to the LF; (2) The LF fuses the measured data with the feedback data from the GF, and then updates the time and filtered information into the latest value; (3) The GF fuses all the corrected data into a new global state estimation, and outputs the global state estimation and meanwhile feeds them back to the LF.

In Figure 1, LF*_i_* takes the latest *Z_i_*, *Q_i_*, *P_i_*, and *X_i_* as input and then independently performs the IAE based on such information. *Z_i_* is the corrected measured data from Lidar or Radar by coordinate transformation and time synchronization. *β* is the information allocation factor of LF*_i_*. *X_g_* is the global optimal estimation. *P_g_* is the V-C matrix of *X_g_*. Then, LF*_i_* sends *Q_i_*, *R_i_*, *X_i_*, and *P_i_* to the GF. *Q_i_* is the system noise V-C matrix of LF*_i_*. *R_i_* is the measurement noise V-C matrix of LF*_i_*. *X_i_* is the state estimated value of LF*_i_*. *P_i_* is the V-C matrix of *X_i_*. At last, GF calculates *Q_i_*, *X_i_*, and *P_i_*, and feeds them back to the LF*_i_*, and finally outputs the optimal result *X_g_* and *P_g_*. When *i* = 1, the data source of LF*_i_* is Lidar, otherwise is Radar for *i* = 2.

### 3.2. Local Kalman Filter

Since vehicle’s transverse and longitudinal velocity changes slowly during driving, so LF can use the linear discrete Kalman filter model, as expressed by [23]:

(1) $$X_{t+1}=\Phi X_{t}+GW_{t}$$

(2) $$Z_{t+1}=HX_{t+1}+V_{t+1}$$

where *X*_*t*+1_ and *X_t_* are the state vector *X* = [*x_s_ x_v_ x_a_ y_s_ y_v_ y_a_*]^T^ at time *t* + 1 and *t*, respectively. *x_s_* is the relative transverse displacement, *x_v_* is the relative transverse velocity, *x_a_* is the relative transverse acceleration, *y_s_* is the relative longitudinal displacement, *y_v_* is the relative longitudinal velocity, and *y_a_* is the relative longitudinal acceleration. *Φ* is the state transition matrix, which can be expressed by:

(3) $$\Phi =\left[\begin{array}{cccccc} 1 & \Delta t & \Delta t^{2}/2 & 0 & 0 & 0\\ 0 & 1 & \Delta t & 0 & 0 & 0\\ 0 & 0 & 1 & 0 & 0 & 0\\ 0 & 0 & 0 & 1 & \Delta t & \Delta t^{2}/2\\ 0 & 0 & 0 & 0 & 1 & \Delta t\\ 0 & 0 & 0 & 0 & 0 & 1 \end{array}\right]$$

where Δ*t* is the time interval of the filtered data. *G* is the coefficient of the system noise matrix and defined as *G* = [Δ*t*^3^/6 Δ*t*^2^/2 Δ*t* Δ*t*^3^/6 Δ*t*^2^/2 Δ*t*]^T^. *W_t_* is the system noise vector at time *t*, *Z*_*t*+1_ is the measurement vector at time *t* + 1, *H* is the coefficient of the measurement matrix, which is a six-order unit matrix, and *V*_*t*+1_ is the measurement noise vector at time *t* + 1. *W_t_* and *V*_*t*+1_ are uncorrelated zero-mean white Gaussian noise sequence with covariance, i.e.:

(4) $$\mathrm{cov}[V_{k},V_{j}]=R_{k}\delta_{kj}$$

(5) $$\mathrm{cov}[W_{k},W_{j}]=Q_{k}\delta_{kj}$$

(6) $$\mathrm{cov}[W_{k},V_{j}]=0$$

(7) $$\delta_{kj}=\{\begin{array}{c} 1,k=j\\ 0,k\neq j \end{array}$$

where the term cov is the function of calculating the covariance matrix, and δ*_kj_* is the dirichlet function. From the above linear discrete system, the CKF working behaviors can be characterized by [23]:

(8) $$X_{t+1/t}=\Phi X_{t/t}+GW_{t}$$

(9) $$P_{t+1/t}=\Phi P_{t/t}\Phi^{Τ}+Q_{t+1}$$

(10) $$K_{t+1}=P_{t+1/t}H^{Τ}{\left[HP_{t+1/t}H^{Τ}+R_{t+1}\right]}^{-1}$$

(11) $$P_{t+1/t+1}=[I-K_{t+1}H]P_{t+1/t}$$

(12) $$X_{t+1/t+1}=X_{t+1/t}+K_{t+1}v_{t}$$

where v*_t_* is the innovation vector, and ${\hat{\text{C}}}_{vt}$ is the V-C matrix of v*_t_*. To improve the real-time performance and ergodicity, we set a sliding estimation window with size *N* for averaging ${\hat{\text{C}}}_{vt}$. Finally, ${\hat{\text{R}}}_{t+1}$ and ${\hat{\text{Q}}}_{t+1}$ can be calculated according to ${\hat{\text{C}}}_{vt}$. So v*_t_*, ${\hat{\text{C}}}_{vt}$, ${\hat{\text{R}}}_{t+1}$, and ${\hat{\text{Q}}}_{t+1}$ are defined by [10]:

(13) $$v_{t}=Z_{t}-HX_{t+1/t}$$

(14) $${\hat{C}}_{v_{t}}=\frac{1}{N}\sum_{j=t-N+1}^{t}v_{j}v_{j}^{Τ}$$

(15) $${\hat{R}}_{t+1}={\hat{C}}_{v_{t}}-HP_{t+1/t}H^{Τ}$$

(16) $${\hat{Q}}_{t+1}=K_{t}{\hat{C}}_{v_{t}}K_{t}^{Τ}+P_{t/t}-\Phi P_{t-1/t-1}\Phi^{Τ}\approx K_{t}{\hat{C}}_{v_{t}}K_{t}^{Τ}$$

If the innovation vector *v_t_* of Lidar includes seriously divergent noise or errors, ${\hat{C}}_{vt}$ is divergent as well, so we use the Chi-square hypothesis test to mitigate this negative influence. The Chi-square hypothesis test can estimate the deviation between the observed and theoretical values of samples. The deviation decides the Chi-square values, i.e., the higher the Chi-square values, the more abnormal the samples, so a certain Chi-square distribution threshold enables us to identify abnormal samples. Considering *v_t_* is the zero-mean white noise with Gaussian distribution, we obtain:

(17) $$U_{t}(m)=\frac{v_{t}^{2}(m)}{C_{v_{t-1}}(m,m)}\sim \chi_{\alpha }^{2}(1)$$

where *v_t_*(*m*) is the *m*th element of vector *v_t_*, *C*_*vt*−1_(*i, i*) is the *i*th diagonal element of matrix ${\hat{C}}_{vt}$, and $\chi_{\alpha }^{2}(1)$ represents a Chi-square distribution with *m* freedom degree and confidential level *α*. If *U_t_*(*m*) < $\chi_{\alpha }^{2}(1)$, the new innovation vector is normal, otherwise it is abnormal. To mitigate the influence of the abnormal innovation vector, the abnormal element is corrected by:

(18) $$v_{t}(m)=\{\begin{array}{cc} v_{t}(m), & 0\leq U_{t}(m)\leq \chi_{\alpha }^{2}(1)\\ v_{t(m)}\exp\left(\frac{-(U_{t}(m)-\chi_{\alpha }^{2}(1))}{\chi_{\alpha }^{2}(1)}\right), & U_{t}(m)\geq \chi_{\alpha }^{2}(1) \end{array},m=1,...,n$$

### 3.3. Global Kalman Filter

To get the optimal estimation, the GF analyzes and integrates the estimated information of the LFs. The computational process of GF is as follows:

Step 1. Optimal information fusion. GF fuses all the state estimations of the local filters into the optimal fusion state vector ${\hat{X}}_{g}$, and meanwhile it fuses the covariance matrix into *P_g_*, as expressed by [9]:

(19) $${\hat{X}}_{g}=P_{g}\sum_{i=1}^{2}P_{i}^{-1}\hat{X_{i}}$$

(20) $$P_{g}=(\sum_{i=1}^{2}P_{i}^{-1}{)}^{-1}$$

Step 2. Calculating the information allocation factor. The information allocation factors *β*_1_ and *β*_2_ are used to adjust *Q_i_*, *P_i_*, and the weight of ${\hat{X}}_{1}$ and ${\hat{X}}_{2}$ in the optimal fusion state ${\hat{X}}_{g}$. *β*_1_ is calculated according to tr(*R*_1_) and tr(*R*_2_). tr(*R*_1_) and tr(*R*_2_) indicate the trace of *R*_1_ and *R*_2_, respectively. *R* can truly reflect the performance of the current filter. Table 1 lists the relation between *R* and the accuracy of Lidar. When the Signal-Noise Ratio (SNR) of Lidar spans from 80 dB to 87 dB, the value of R_1_ decreases from 0.0021 to 0.0004, and accordingly the accuracy declines from 0.0362 to 0.0164. This implies that there exists a directly proportional relationship between these two quantities. If the deteriorating environment makes the accuracy of Lidar drop down to a certain threshold such as tr(*R*_1_) ≥ 20 tr(*R*_2_), we shall choose *β*_1_ << 1, so as that the output of GF can approximate to the output of only Radar available. Generally speaking, Lidar is almost ten times as accurate as the Radar, so when tr(*R*_1_) ≥ 20 tr(*R*_2_), the accuracy of Radar becomes far better than that of Lidar, i.e., the Lidar already loses effectivity and produces divergent results. Therefore, *β*_1_ is defined by:

(21) $$\beta_{1}=\{\begin{array}{ll} 0.01, & tr(R_{1})\geq 20tr(R_{2})\\ \frac{1/tr(R_{1})}{1/tr(R_{1})+1/tr(R_{2})}, & tr(R_{1})<20tr(R_{2}) \end{array}$$

According to the principle of information distribution conservation *β*_1_ + *β*_2_ = 1 [9], so *β*_2_ is expressed by:

(22) $$\beta_{2}=1-\beta_{1}$$

Step 3. Filtering state feedback. GF uses *β*_1_ and *β*_2_ to calculate the latest system information that will be feed back to the LFs, as expressed by:

(23) $$Q^{-1}=Q_{1}^{-1}+Q_{2}^{-1}\;Q_{i}^{-1}=\beta_{i}Q^{-1},\;i=1,\;2$$

(24) $$P^{-1}=P_{1}^{-1}+P_{2}^{-1}\;P_{i}^{-1}=\beta_{i}P^{-1},\;i=1,\;2$$

(25) $$\hat{X_{i}}=\hat{X_{g}},\;i=1,\;2$$

### 3.4. Coordinate Transformation

**Figure 2:**
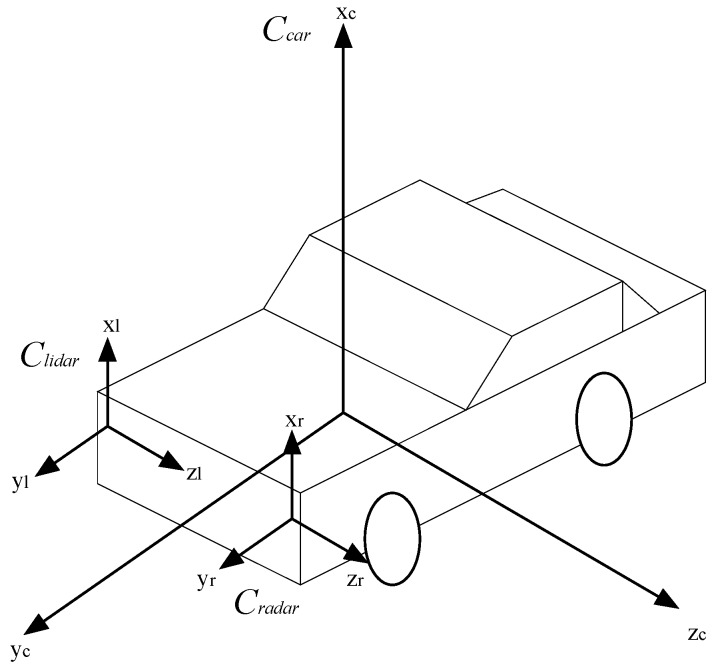
The relations among *C_lidar_*, *C_radar_*, and *C_car_*.

The collected motion state of the forward vehicle should be placed into the current car’s coordinate system, but in fact, the data collected by Lidar and Radar are based on their own coordinate systems, so in order to realize spatial synchronization of the measured data, we propose a method to transform the Lidar coordinate system *C_lidar_*(*x_l_*, *y_l_*, *z_l_*) and the Radar coordinate system *C_radar_*(*x_r_*, *y_r_*, *z_r_*) into the current car coordinate system *C_car_*(*x_c_*, *y_c_*, *z_c_*). Figure 2 illustrates the relations between these coordinate systems.

First, we implement the coordinate transformation between *C_lidar_* and *C_car_*. Suppose *P^lidar^* = (*x^lidar^*, *y^lidar^*, *z^lidar^*)^T^ is a point in *C_lidar_*, and *P^car^* = (*x^car^*, *y^car^*, *z^car^*)^T^ is the corresponding point in *C_car_*, so their coordinate transformation can be achieved by:

(26) $$P^{car}=R_{lidar\to car}*P^{lidar}+T_{lidar\to car}$$

where *T_lidar→car_* is the mounted coordinate of Lidar onto the coordinate system *C_car_*, which can be simply measured. *R_lidar→car_* is the rotation matrix in *C_lidar_* relative to *C_car_*. In *R_lidar→car_*, the pitch angle *α* can be obtained by rotating around the *x*-axis, and similarly, the deflection angle *φ* can be obtained by rotating around the *z*-axis, so *R_lidar→car_* is defined by:

(27) $$R_{lidar\to car}=\left[\begin{array}{ccc} \cos\varphi & -\sin\varphi \cos\alpha & \sin\varphi \cos\alpha \\ \sin\varphi & \cos\varphi \cos\alpha & -\cos\varphi \cos\alpha \\ 0 & \sin\alpha & \cos\alpha \end{array}\right]$$

More details about the calculation of *α* and *φ* can be found in [24]. Similarly, we can obtain *T_radar→car_* and *R_radar→car_* to execute the coordinate transformation between *C_radar_* and *C_car_*, and thus achieving the spatial synchronization of Lidar and Radar.

### 3.5. Time Synchronization

Since various sensors have different data sampling frequenci, the time error calibration has to be considered. To improve the accuracy of data fusion, we choose an appropriate time slice as the time interval of data fusion. Since the sampling frequency of Lidar is higher than that of Radar, the collected data from Lidar need to be calculated in accordance with the Radar by interpolation and extrapolation. Suppose the data sampling interval of Radar as the time point of data fusion *t_i_*, so we need to find a corresponding time point in data sampling interval of Lidar as interpolate point, and then calculating the measured value of Lidar at time *t_i_* through Lagrange three-point interpolation.

Assume the measured data sequence from Lidar is *X*_*i*−1_, *X_i_*, and *X*_*i*+1_ at time *t*_*mi*−1_, *t_mi_*, and *t*_*mi*+1_, and *t_mi_* − *t*_*mi*−1_ = *t_mi_* − *t*_*mi*+1_ = *h*. *t_mi_* ≤ *t_i_* ≤ *t*_*mi*+1_, i.e., *t_i_* = *t_mi_* + *τh*, 0 ≤ *τ* ≤ 1, so the estimated value ${\bar{X}}_{i}$ at time *t_i_* is defined by:

(28) $$\bar{X_{i}}=\frac{\left(t_{i}-t_{m_{i}})(t_{i}-t_{m_{i+1}}\right)}{\left(t_{m_{i-1}}-t_{m_{i}})(t_{m_{i-1}}-t_{m_{i+1}}\right)}X_{i-1}+\frac{\left(t_{i}-t_{m_{i-1}})(t_{i}-t_{m_{i+1}}\right)}{\left(t_{m_{i}}-t_{m_{i-1}})(t_{m_{i}}-t_{m_{i+1}}\right)}X_{i}+\frac{\left(t_{i}-t_{m_{i-1}})(t_{i}-t_{m_{i}}\right)}{\left(t_{m_{i+1}}-t_{m_{i-1}})(t_{m_{i+1}}-t_{m_{i}}\right)}X_{i+1}$$

## 4. Results

### 4.1. Simulation

**Figure 3:**
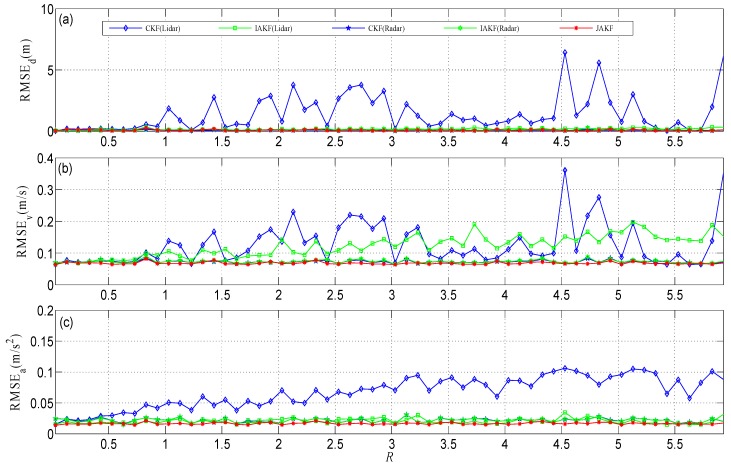
Comparisons of motion state estimation against different noise variances *R*: (**a**) The displacement estimation; (**b**) The velocity estimation; (**c**) The acceleration estimation.

**Figure 4:**
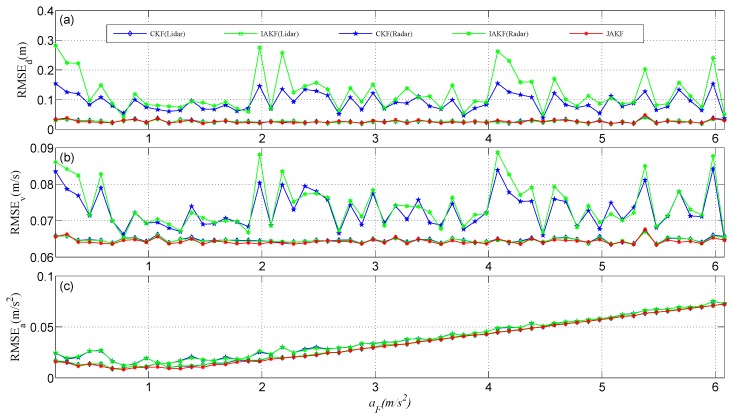
Comparisons of motion state estimation against different accelerations *a_F_*: (**a**) The displacement estimation; (**b**) The velocity estimation; (**c**) The acceleration estimation.

We conducted the simulation using MATLAB R2014a on a computer equipped with an Intel Core i7-4790 CPU (3.60 GHz) and Windows 64. There are two important factors that can affect the accuracy of the filtered results. At the one hand, the noise intensity is treated as a prevailing concern and can be quantified by the variance *R*. In the simulation, the Lidar noise variance is increased by 0.1 per time span from 0.03 to 5.93, while the Radar noise variance maintains its initial value of 0.1. The initial velocity, acceleration, and displacement of the forward vehicle are 2 m/s, 0.18 m/s^2^, and 0 m, respectively. Figure 3 shows the root mean squared error (RMSE), where JAKF produces the maximum RMSE of the displacement 0.2323 m and the average 0.0818 m, the maximum RMSE of the velocity 0.0837 m/s and the average 0.0689 m/s, and the maximum RMSE of the acceleration 0.0237 m/s^2^ and the average 0.0171 m/s^2^. When the Lidar noise variance is increased to 0.93, the RMSEs of the displacement, velocity, and acceleration produced by CKF (Lidar) increase to 10-fold, so the results of CKF (Lidar) have diverged. Although IAKF (Lidar) can adapt to the increased noise intensity, the RMSE of JAKF is significantly smaller than that of IAKF (Lidar). At the other hand, the acceleration *a_F_* of the forward vehicle in the CA model is another matter. In the simulation, *a_F_* is increased by 0.1 m/s^2^ per time from 0.18 m/s^2^ to 6.08 m/s^2^ and the noise variances of Lidar and Radar are fixed at 0.03 and 0.1, respectively. Figure 4 compares the motion state estimation against different acceleration values *a_F_*. JAKF contributes the maximum RMSE of the displacement 0.0403 m and the average 0.0261 m, the maximum RMSE of the velocity 0.0664 m/s and the average 0.0643 m/s, and the maximum RMSE of the acceleration 0.0737 m/s^2^ and the average 0.0346 m/s^2^. In the increased acceleration situation, the RMSEs of CKF (Radar) and IAKF (Radar) are larger than the RMSEs of CKF (Lidar), IAKF (Lidar), and JAKF.

Regarding the comparative results in Figure 3 and Figure 4, we can conclude: (i) JAKF enables bounding the RMSE of the motion state estimation to an acceptable level, so the JAKF is robust; (ii) JAKF outputs the smallest RMSE of the motion state estimation, so the JAKF is more accurate than CKF and IAKF; (iii) JAKF uses multi-sensors data fusion to realize the global adaptation, so JAKF can be more adaptive than the single-sensor adaptive filter through performance compensation between different sensors.

### 4.2. Experiment

To obtain accurate data, the test car is equipped with a high precision URG-04LX Lidar developed by Hokuyo (Osaka, Japan), and a millimeter-wave Radar ESR, developed by Delphi (Warren, OH, USA). The forward car collects the displacement, velocity, and acceleration from a Controller Area Network (CAN) bus as a benchmark that is used to compare with the filtered results to evaluate JAKF. CAN bus is a vehicle bus standard designed to allow microcontrollers and devices to communicate with each other in applications. The motion states of vehicles can be easily obtained from some vehicle-sensors by CAN bus. CAN bus even can correct the current velocity and direction. Table 2 lists the parameters of URG-04LX and ESR. 

**Figure 5:**
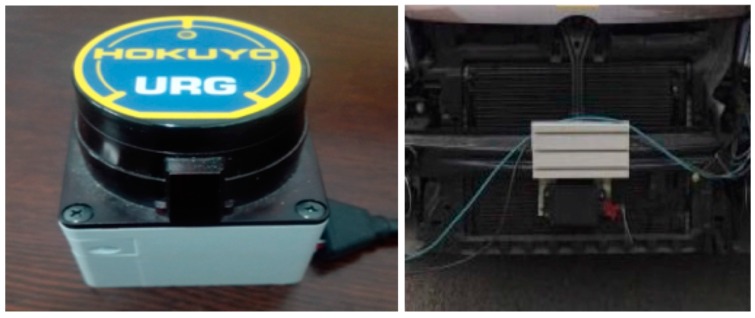
The URG-04LX Lidar (**Left**) and the ESR Radar (**Right**).

**Figure 6:**
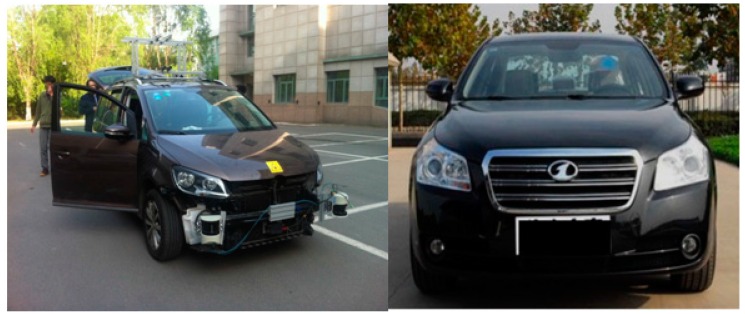
The test car at the left side and the forward car at the right side.

**Figure 7:**
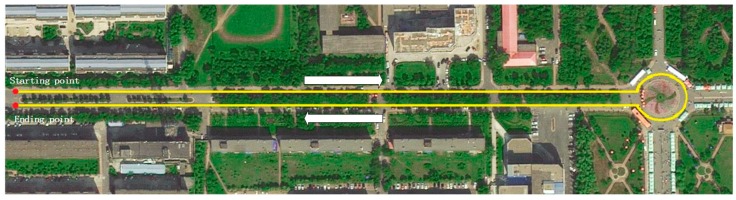
The experimental scenario and route.

Figure 5 shows the employed URG-04LX and ESR in the experiments, respectively. Figure 6 shows the test car and the forward car, respectively. Figure 7 shows the experimental scenario and route.

In the experiments, *R*_1_, *R*_2_, *Q*_1_ and *Q*_2_ represent the noises of local filters LF1 and LF2, and their initial values are 0.03, 0.1, 0.000001 and 0.000001, respectively. R_1_ and R_2_ are measurement noise covariance in Kalman filter model and are calculated by IAE at each moment. *Q*_1_ and *Q*_2_ are constant and indicate the systemic noise covariance in Kalman filter model. The sliding window size of the innovation sequence is 30, and the given confidential level *α* is 0.005 in Chi-square distribution, so $X_{0.005}^{2}$(1) = 7.879. We conducted three experiments to evaluate the JAKF results. In the first two experiments, the forward car moves along a straight line at a constant and varying acceleration, respectively. In the third, the forward car is permitted to change lanes and thus produces obvious displacement in the lateral.

In the first experiment, the forward car just shifts visibly in the vertical, so we only focus on the longitudinal motion information. The initial longitudinal velocity, acceleration, and displacement of the forward vehicle are 5.43 m/s, 0.18 m/s^2^, and 0 m, respectively. The test car follows the forward car so as to ensure sensors can detect the forward car. We collected 5000 samples with frequency 100 Hz.

**Figure 8:**
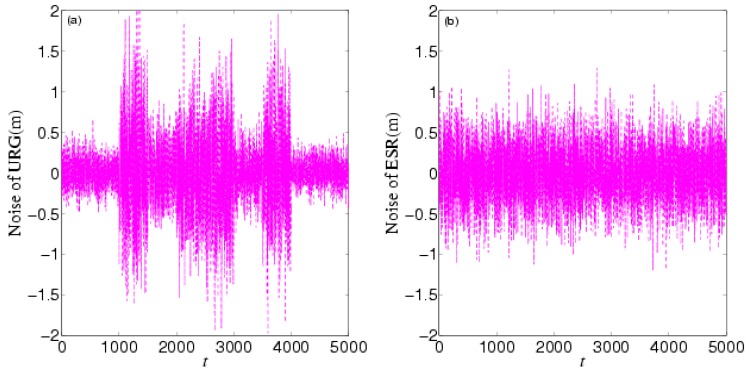
The noise of (**a**) URG and (**b**) ESR in the first experiment.

**Figure 9:**
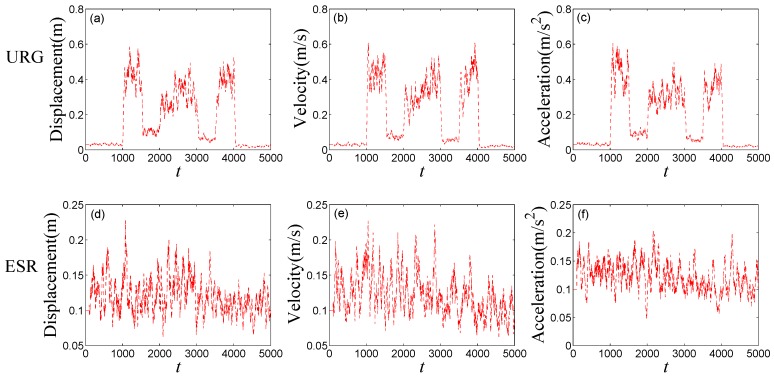
The measurement noise V-C matrix *R* of the local-filter output of URG and ESR in the first experiment (The R of displacement of (**a**) URG and (**d**) ESR, the R of velocity of (**b**) URG and (**e**) ESR and the R of acceleration of (**c**) URG and (**f**) ESR).

Figure 8 shows the noises of URG and ESR, respectively. URG is suffered from the continuous high noise within the 1000th~1500th time slots, the 2000th~3000th time slots, and the 3500th~4000th time slots, while the noise of ESR always holds steady, but URG has less error than ESR when working well during the 1500th~2000th, 3000th~3500th, and the 4000th~5000th time slots. Figure 9 provides the measurement noise V-C matrix *R* of the local-filter output of URG and ESR, which indicates the *R* of JAKF can adapt to the variation of the noise.

**Figure 10:**
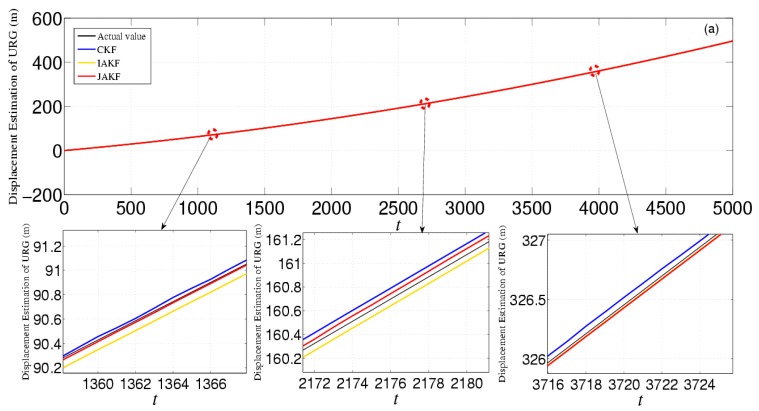

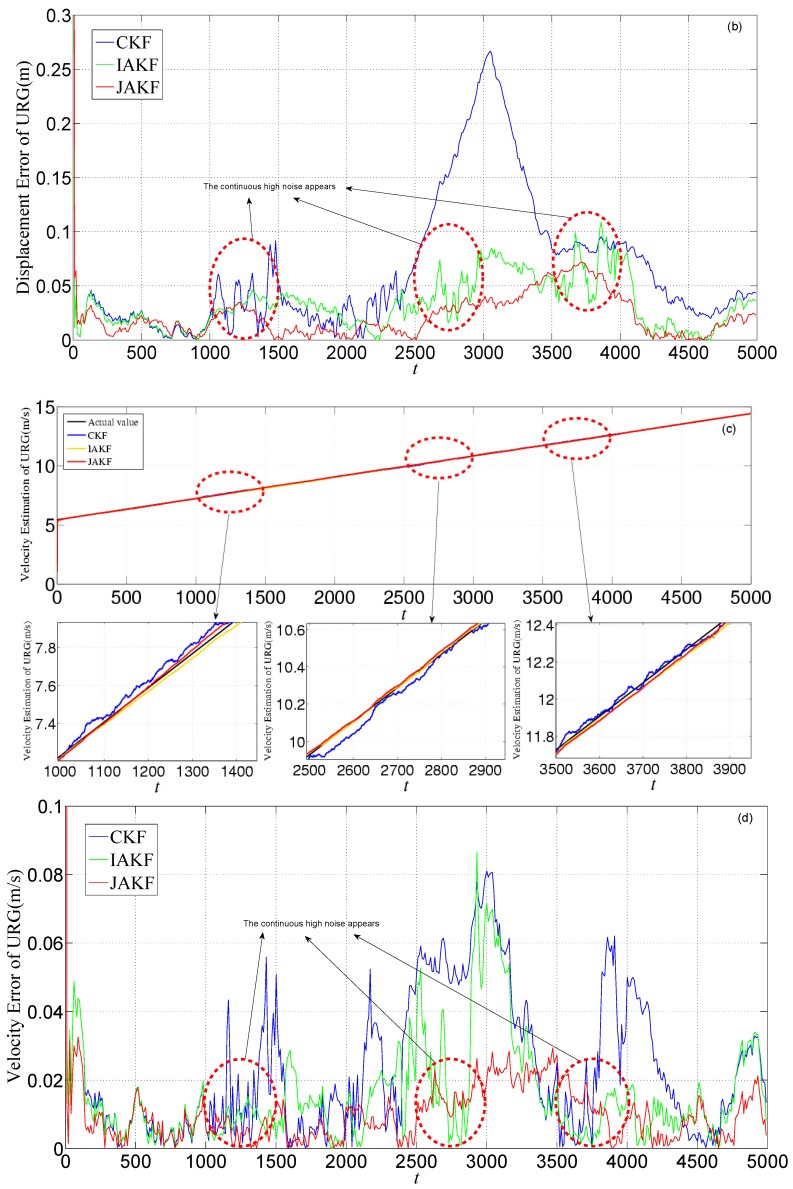

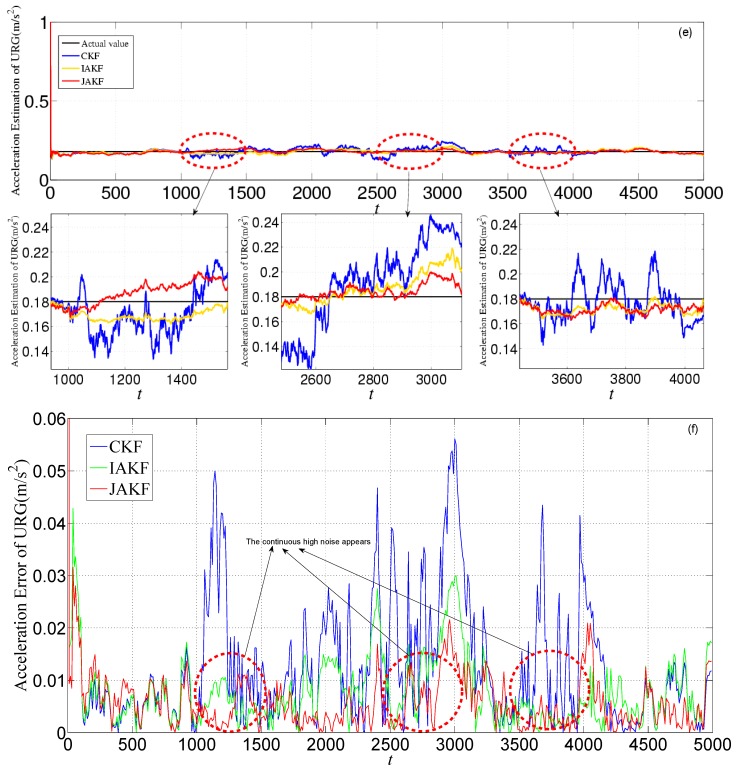
Estimation and error comparison of URG in the first experiment: (**a**) Displacement estimation of URG; (**b**) Displacement error of URG; (**c**) Velocity estimation of URG; (**d**) Velocity error of URG; (**e**) Acceleration estimation of URG; (**f**) Acceleration error of URG.

Figure 10 provides the estimation and error comparison of URG. The estimation of error is the abs value of the difference between the actual and estimation values in the same time slot. After the 500th time slot, all the focused three filters converge to a stable state. When the suffered measure noise by URG is normal, these three filtering algorithms can output the normal results. But when the continuous high noise appears during the 1000th~1500th, 2500th~3000th, and 3500th~4000th time slots, the noise variance *R* of URG is increased. In this case, the CKF is incapable of dealing with the continuous serious disturbance, while the JAKF still can produce higher accuracy than CKF and IAKF. At the 3051th time slot, the absolute error of the longitudinal displacement is about 0.2665 m in CKF, while 0.0826 m in IAKF and 0.0392 m in JAKF. At the 3023th time slot, the error of the longitudinal velocity is about 0.0826 m/s in CKF, and 0.0725 m/s in IAKF, while 0.0222 m/s in JAKF. At the 3001th time slot, the error of the longitudinal acceleration is about 0.0560 m/s^2^ in CKF, while 0.0298 m/s^2^ in IAKF, and 0.0148 m/s^2^ in JAKF.

**Figure 11:**
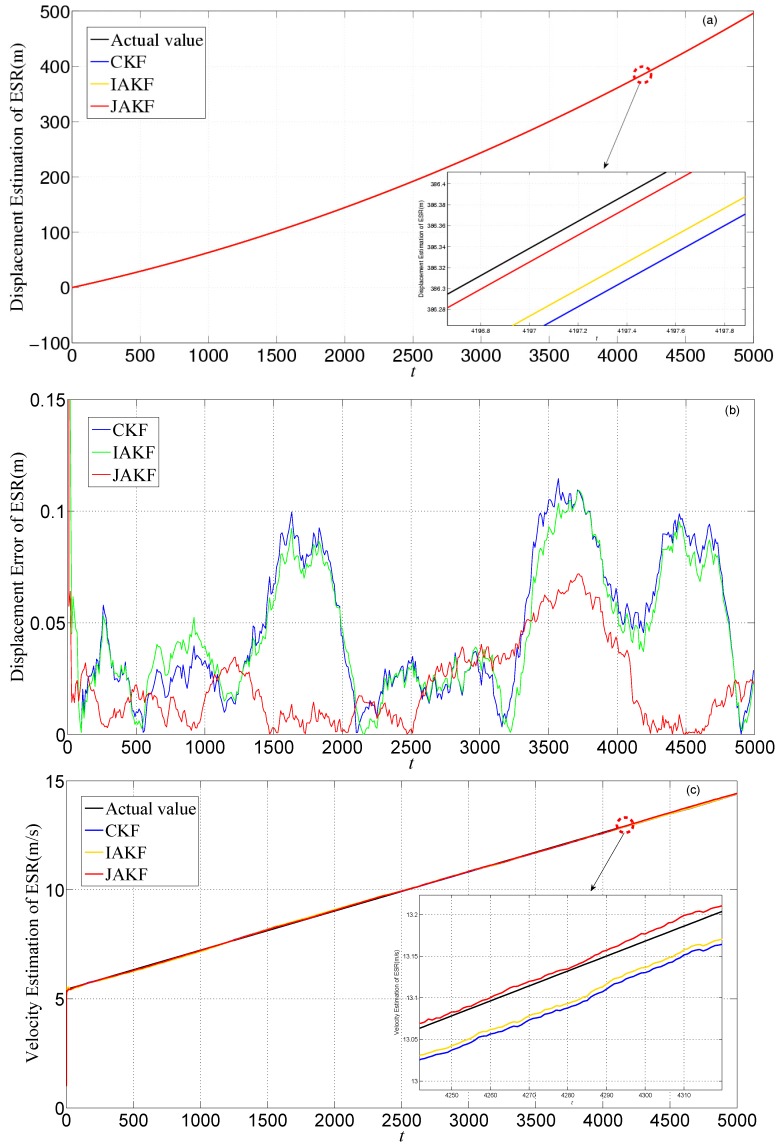

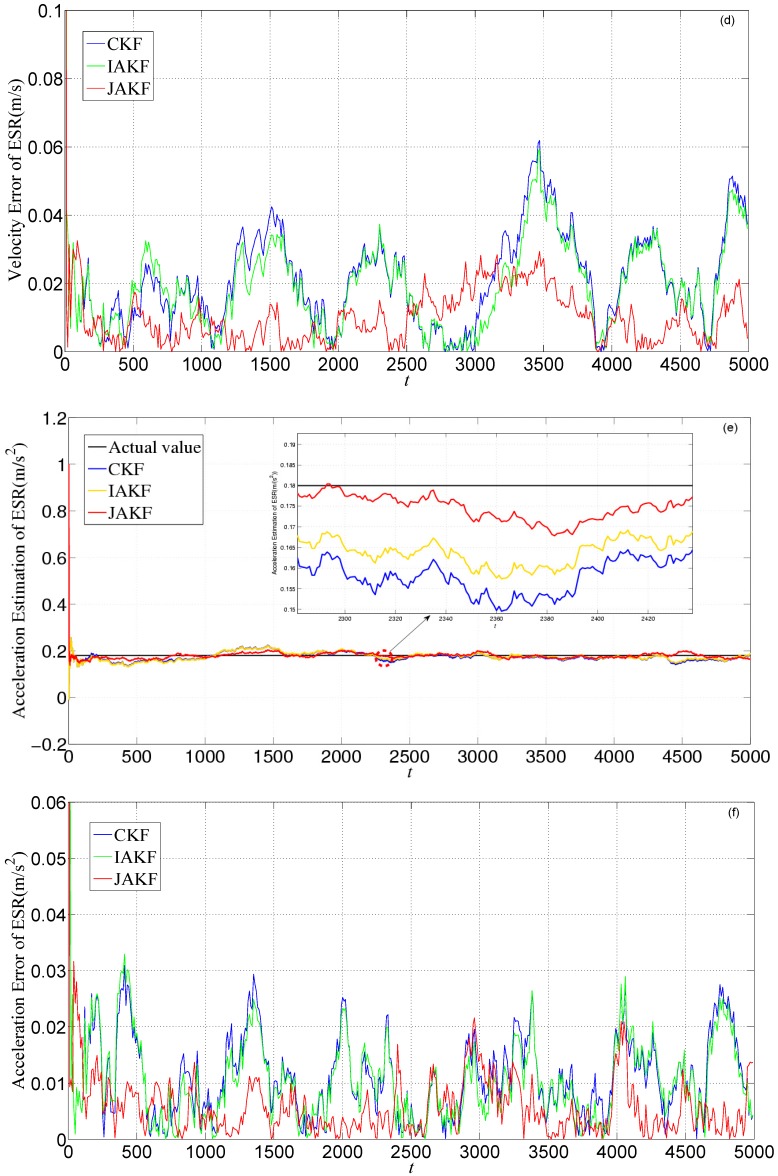
Estimation and error comparison of ESR in the first experiment: (**a**) Displacement estimation of ESR; (**b**) Displacement error of ESR; (**c**) Velocity estimation of ESR; (**d**) Velocity error of ESR; (**e**) Acceleration estimation of ESR; (**f**) Acceleration error of ESR.

Figure 11 gives the estimation and error comparison of ESR. After the filters converged, all the measured noises of the three filters fluctuate within the theoretical range. The errors in CKF and IAKF are nearly similar, while the JAKF still behaves best w.r.t. accuracy. At the 3571th time slot, the error of the longitudinal displacement is about 0.0649 m in JAKF, while 0.1147 m in CKF and 0.1034 m in IAKF. At the 3466th time slot, the error of the longitudinal velocity is about 0.0626 m/s in CKF, and 0.0605 m/s in IAKF, while 0.0289 m/s in JAKF. At the 4776th time slot, the error of the longitudinal acceleration is about 0.0046 m/s^2^ in JAKF, while 0.0297 m/s^2^ in CKF and 0.0257 m/s^2^ in IAKF.

Table 3 lists the comparisons of CKF, IAKF and JAKF about root-mean-square error, maximum error, and variance of filtered results. In Table 3, the RMS error and the variance of JAKF is smaller than the ones of CKF and IAKF, so JAKF has better stability and fault-tolerance against the continuous varying noise, and the accuracy of JAKF is higher than that of the single-sensor filter in the situation where the acceleration of the forward car is constant.

In the second experiment, the forward car moved along a straight line at a varying acceleration. As the first experiment, we only focus on the longitudinal motion in this situation. The initial longitudinal velocity, acceleration, and displacement of the forward vehicle are 0.15 m/s, 0 m/s^2^, and 0 m, respectively. The test car follows the forward car so as to ensure sensors can detect the forward car. We collected 5000 samples with frequency 100 Hz.

**Figure 12:**
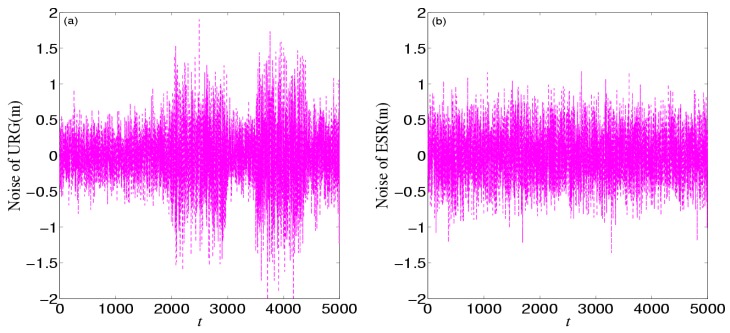
The noise of (**a**) URG and (**b**) ESR in the second experiment.

**Figure 13:**
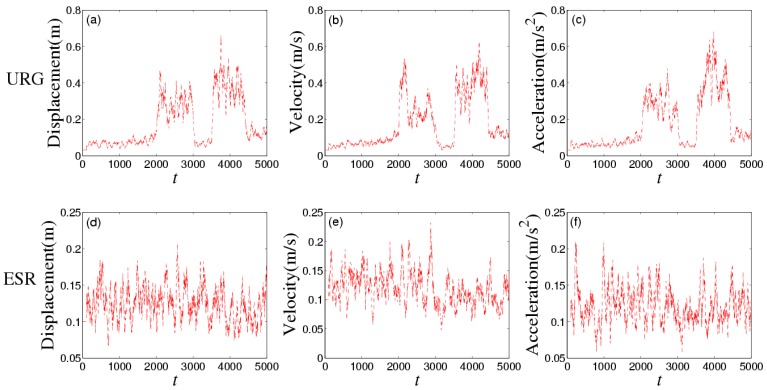
The measurement noise V-C matrix *R* of the local-filter output of URG and ESR in the second experiment (The R of displacement of (**a**) URG and (**d**) ESR, the R of velocity of (**b**) URG and (**e**) ESR and the R of acceleration of (**c**) URG and (**f**) ESR).

Figure 12 shows the noises of URG and ESR. URG suffered from the continuous high noise within the 2000th~3000th time slots, and the 3500th~4400th time slots, while the noise of ESR still holds steady all the time. Figure 13 provides the measurement noise V-C matrix *R* of the local-filter output of URG and ESR.

**Figure 14:**
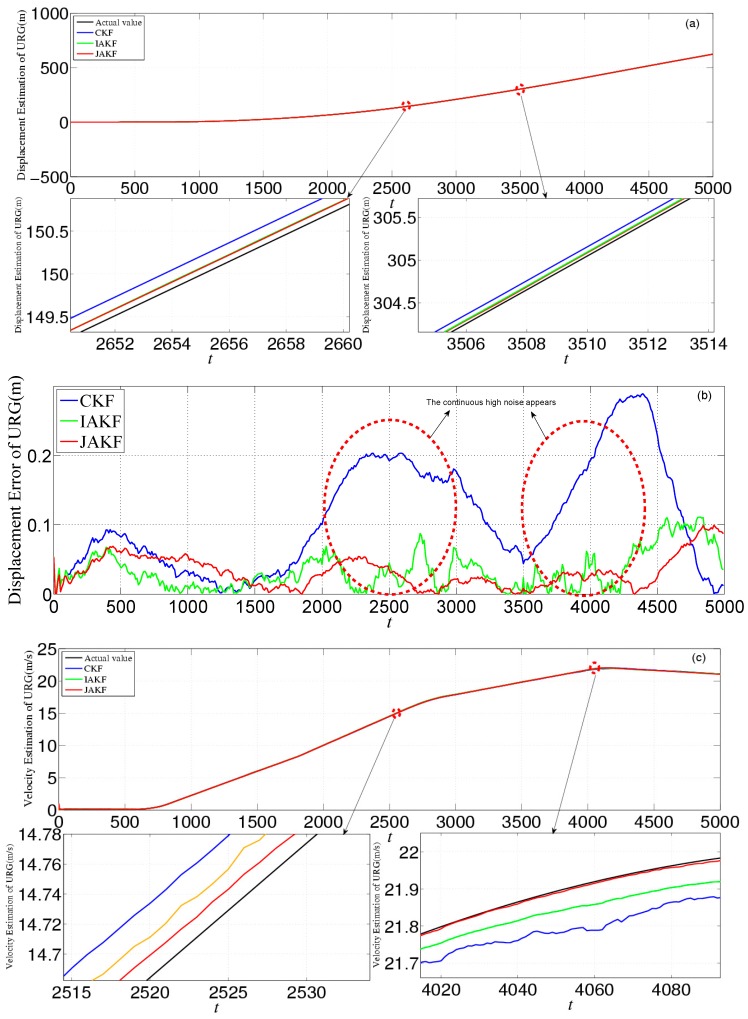

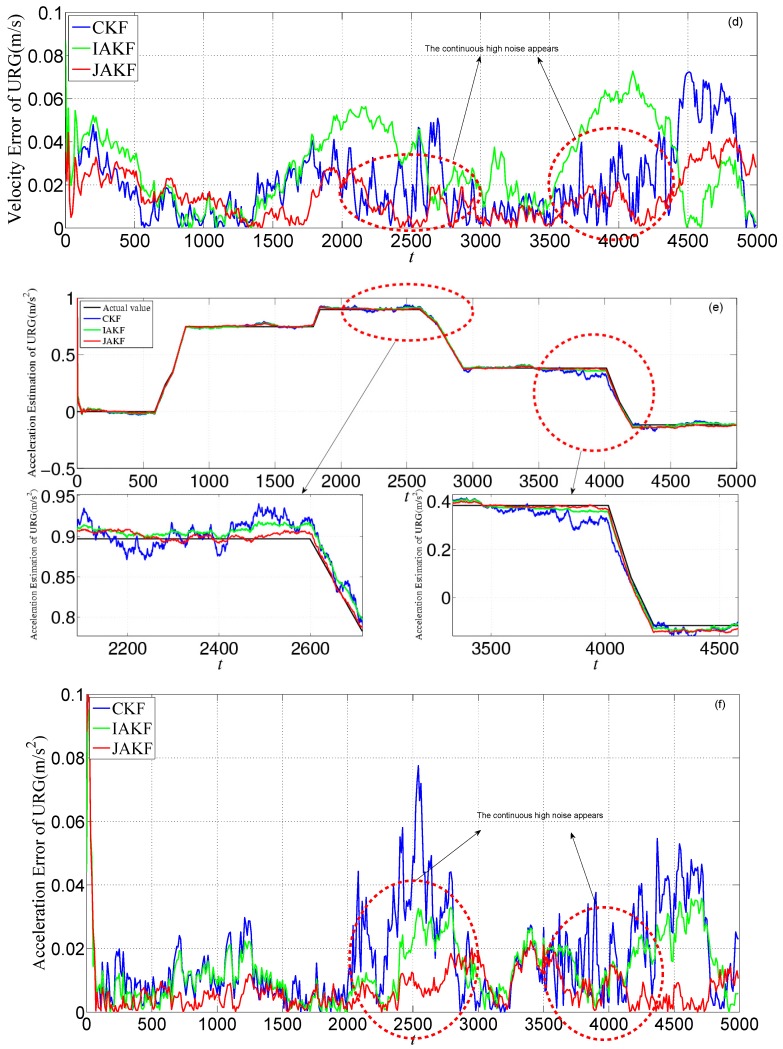
Estimation and error comparison of URG in the second experiment: (**a**) Displacement estimation of URG; (**b**) Displacement error of URG; (**c**) Velocity estimation of URG; (**d**) Velocity error of URG; (**e**) Acceleration estimation of URG; (**f**) Acceleration error of URG.

Figure 14 provides the estimation and error comparison of URG. The continuous high noise appears during the 2000th~3000th time slots and the 3500th~4400th time slots, during which the CKF is badly divergent while the JAKF maintains convergent. For example, at the 2712th time slot, the absolute error of the longitudinal displacement is about 0.1486 m in CKF, while 0.0998 m in IAKF and 0.0152 m in JAKF; the error of the longitudinal velocity is about 0.0513 m/s in CKF, and 0.0226 m/s in IAKF, while 0.0185 m/s in JAKF; and the error of the longitudinal acceleration is about 0.0486 m/s^2^ in CKF, while 0.0375 m/s^2^ in IAKF and 0.0151 m/s^2^ in JAKF.

**Figure 15:**
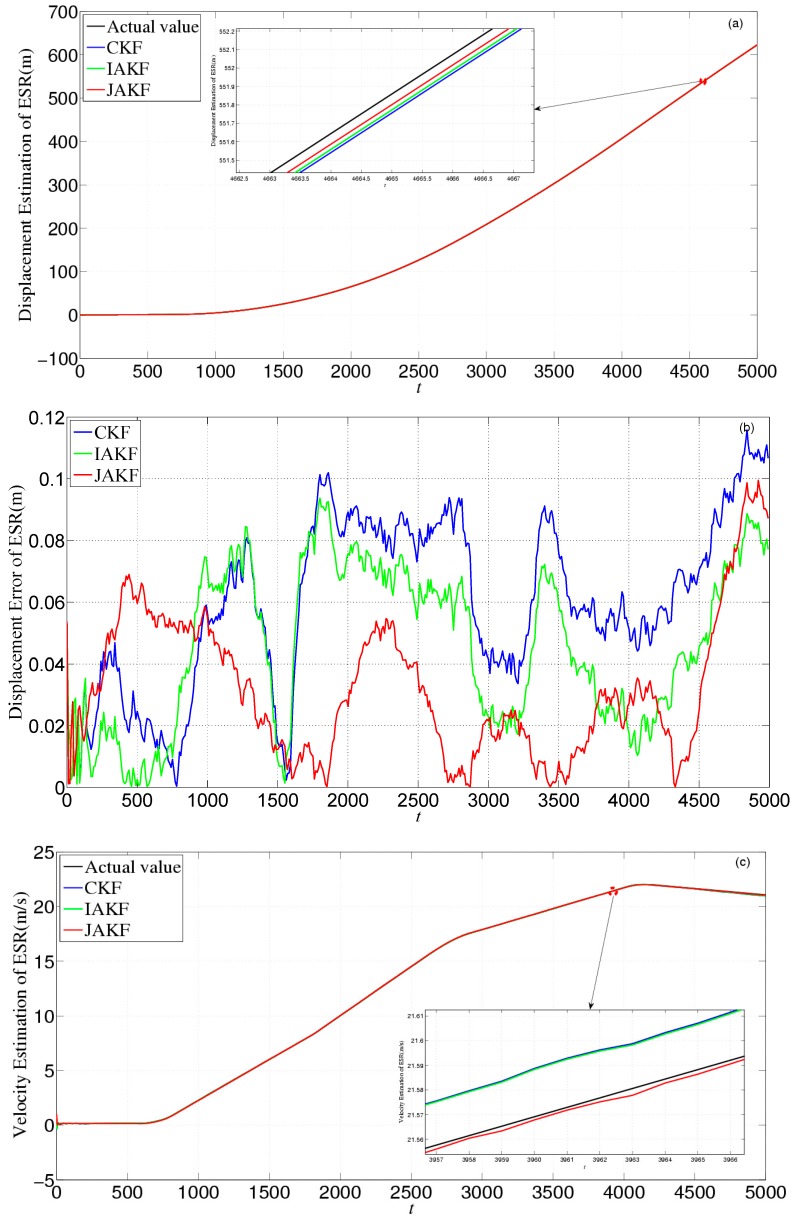

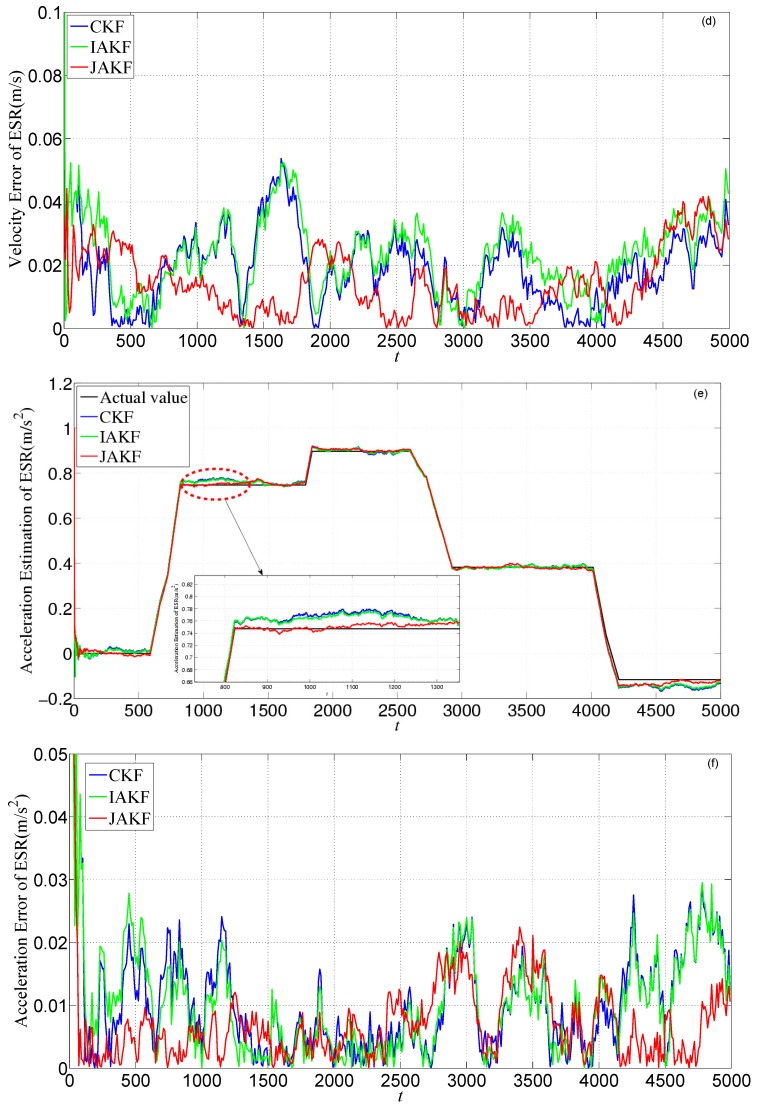
Estimation and error comparison of ESR in the second experiment: (**a**) Displacement estimation of ESR; (**b**) Displacement error of ESR; (**c**) Velocity estimation of ESR; (**d**) Velocity error of ESR; (**e**) Acceleration estimation of ESR; (**f**) Acceleration error of ESR.

Figure 15 gives the estimation and error comparison of ESR. After the filters converged, all the measured noises of the three filters fluctuate within the theoretical range. The errors in CKF and IAKF are still nearly similar while the JAKF behaves best w.r.t. accuracy during most time slots. At the 2256th time slot, the error of the displacement is about 0.0482 m in JAKF, while 0.0883 m in CKF and 0.0752 m in IAKF; the error of the velocity is about 0.0326 m/s in CKF, and 0.0305 m/s in IAKF, while 0.0189 m/s in JAKF; and the error of the acceleration is about 0.0046 m/s^2^ in JAKF, while 0.062 m/s^2^ in CKF and 0.057 m/s^2^ in IAKF.

Table 4 lists the comparisons of CKF, IAKF and JAKF about root-mean-square error, maximum error, and variance of filtered results.

In Table 4, JAKF has high accuracy and stability even though the motion model of the forward car is dynamic. In a nutshell, a varying acceleration imposes little influence on JAKF.

In the third experiment, the forward car is permitted to change lanes and thus produces obvious displacement in the lateral, so we mainly focus on the transverse motion of the forward car in this situation. The initial transverse velocity, acceleration, and displacement of the forward vehicle are 0 m/s, 0 m/s^2^, and 0 m, respectively. During the whole experiment, the test car moves along a straight line and keeps the inter-distance about 3 m~8 m apart from the forward car so as to ensure sensors can detect the forward car. We collected 1000 samples with frequency 100 Hz. We collected the transverse vehicle motion information to CKF, IAKF and JAKF, and correspondingly compared the transverse results with the former two longitudinal ones.

**Figure 16:**
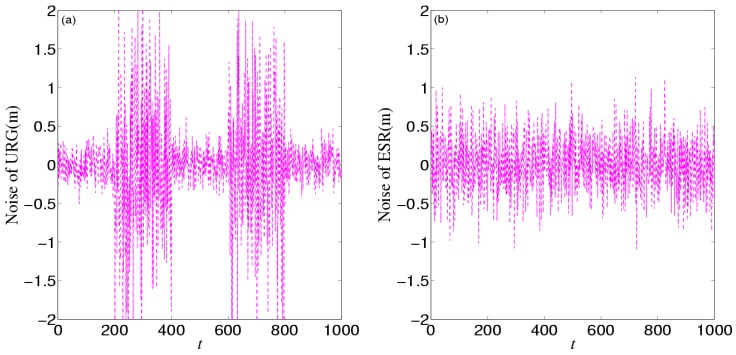
The noise of (**a**) URG and (**b**) ESR in the third experiment.

**Figure 17:**
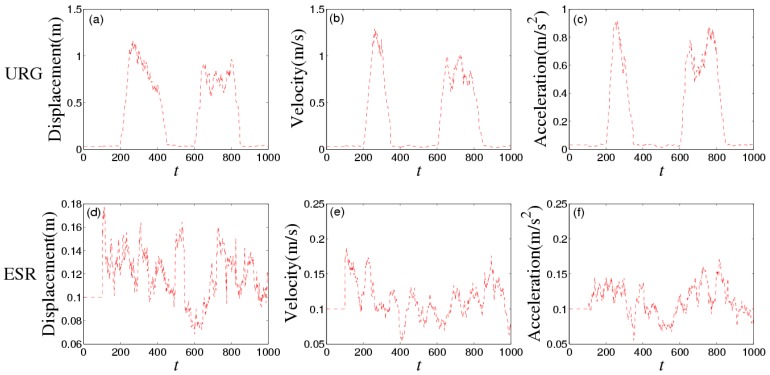
The measurement noise V-C matrix *R* of the local-filter output of URG and ESR in the third experiment: The R of displacement of (**a**) URG and (**d**) ESR; the R of velocity of (**b**) URG and (**e**) ESR and the R of acceleration of (**c**) URG and (**f**) ESR.

Figure 16 shows the noises of URG and ESR. URG is suffered from the continuous high noise within the 200th~400th time slots, and the 600th~800th time slots, while the noise of ESR still holds steady. Figure 17 provides the measurement noise V-C matrix *R* of the local-filter output of URG and ESR.

**Figure 18:**
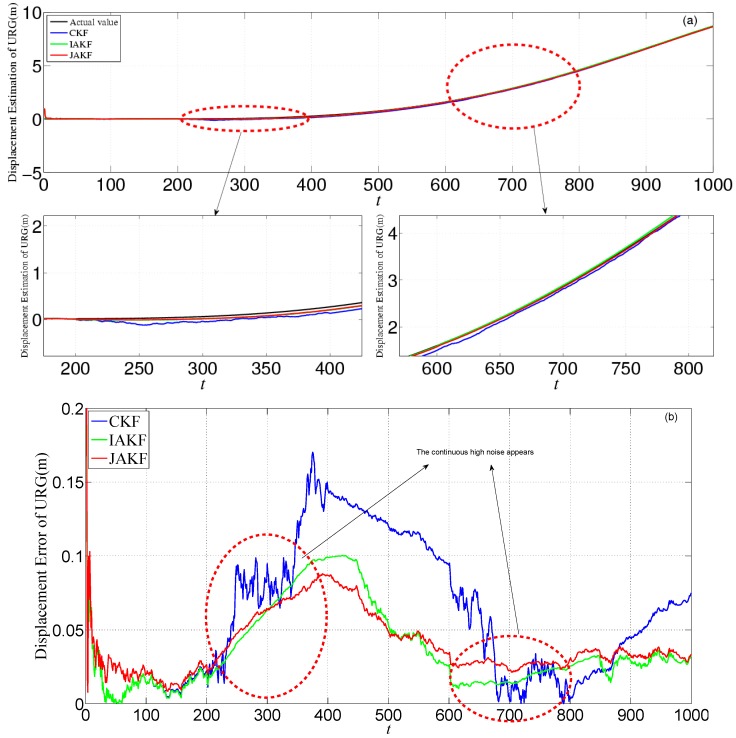

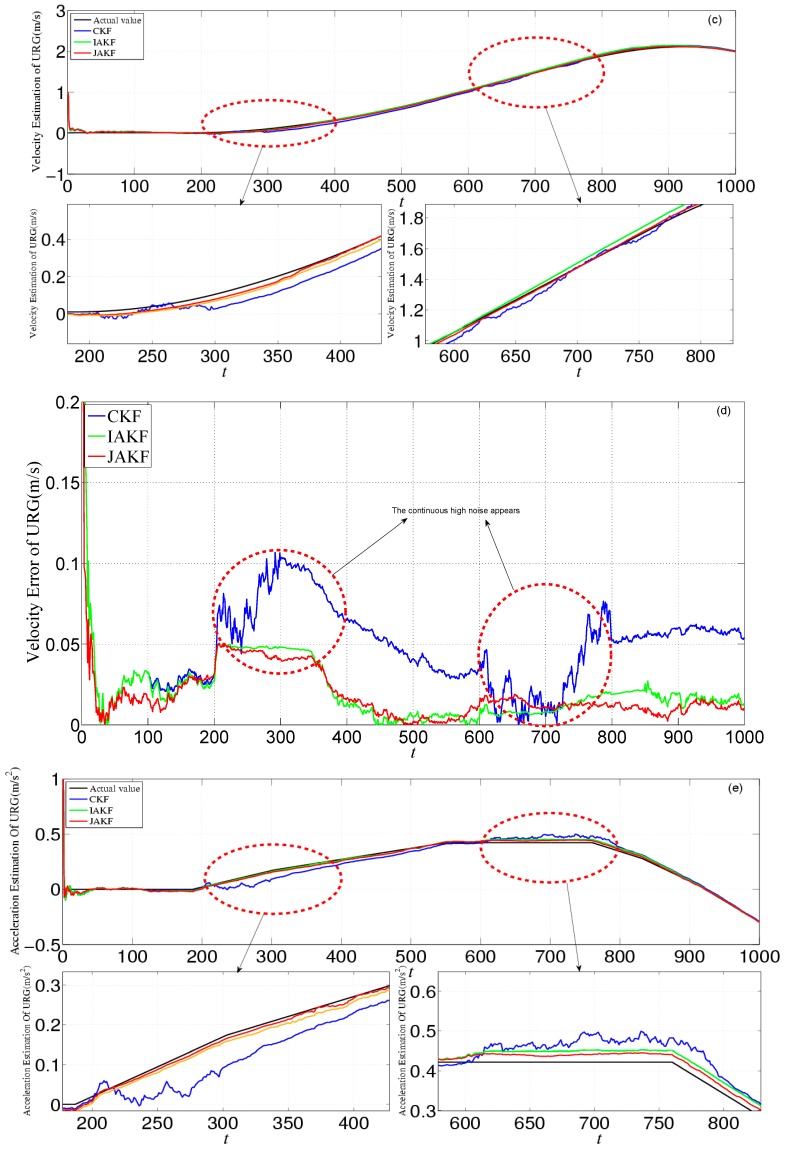

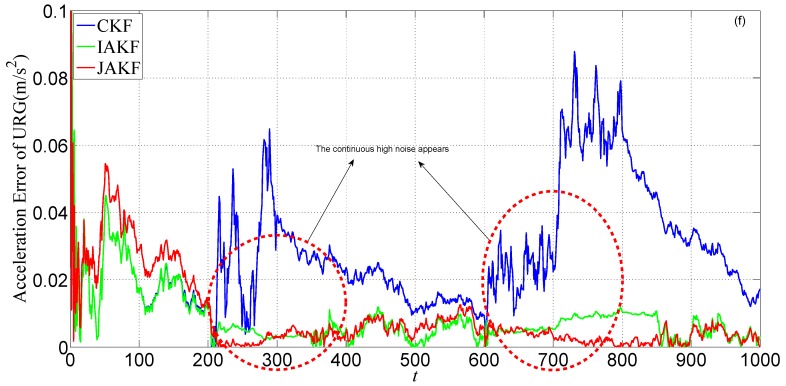
Estimation and error comparison of URG in the third experiment: (**a**) Displacement estimation of URG; (**b**) Displacement error of URG; (**c**) Velocity estimation of URG; (**d**) Velocity error of URG; (**e**) Acceleration estimation of URG; (**f**) Acceleration error of URG.

Figure 18 provides the estimation and error comparison of URG. The continuous high noise appears during the 200th~400th time slots and the 600th~800th time slots, during which the CKF fails to deal with it, while the JAKF maintains convergent. For example, at the 372th time slot, the error of the longitudinal displacement is about 0.178 m in CKF, while 0.0995 m in IAKF and 0.0852 m in JAKF; the error of the longitudinal velocity is about 0.1062 m/s in CKF and 0.0481 m/s in IAKF, while 0.0423 m/s in JAKF; and the error of the longitudinal acceleration is about 0.0341 m/s^2^ in CKF, while 0.0105 m/s^2^ in IAKF and 0.01 m/s^2^ in JAKF.

**Figure 19:**
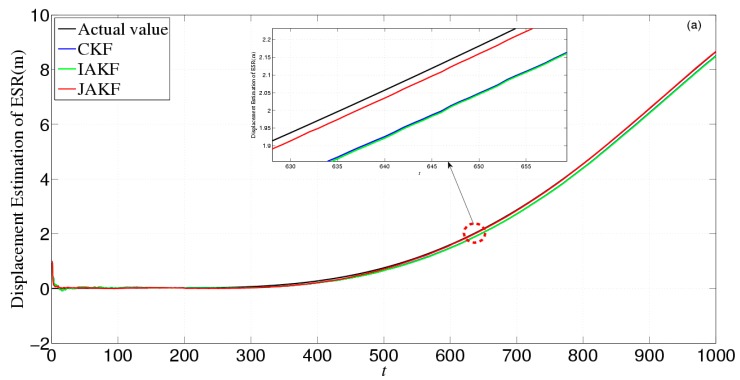

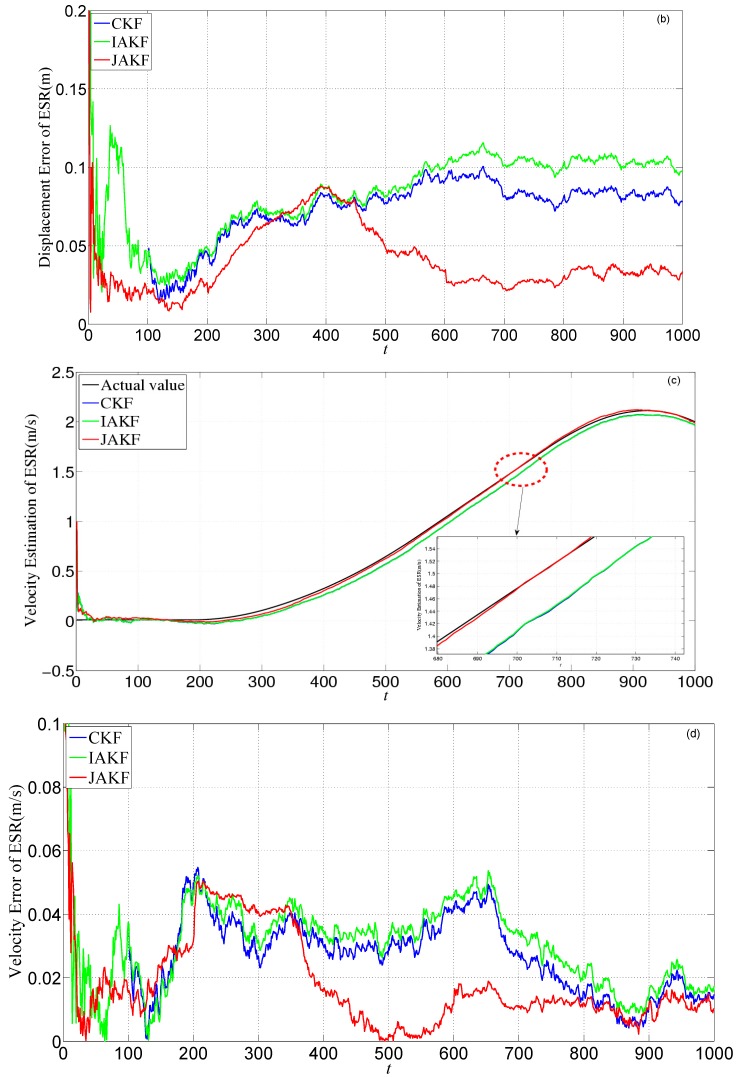

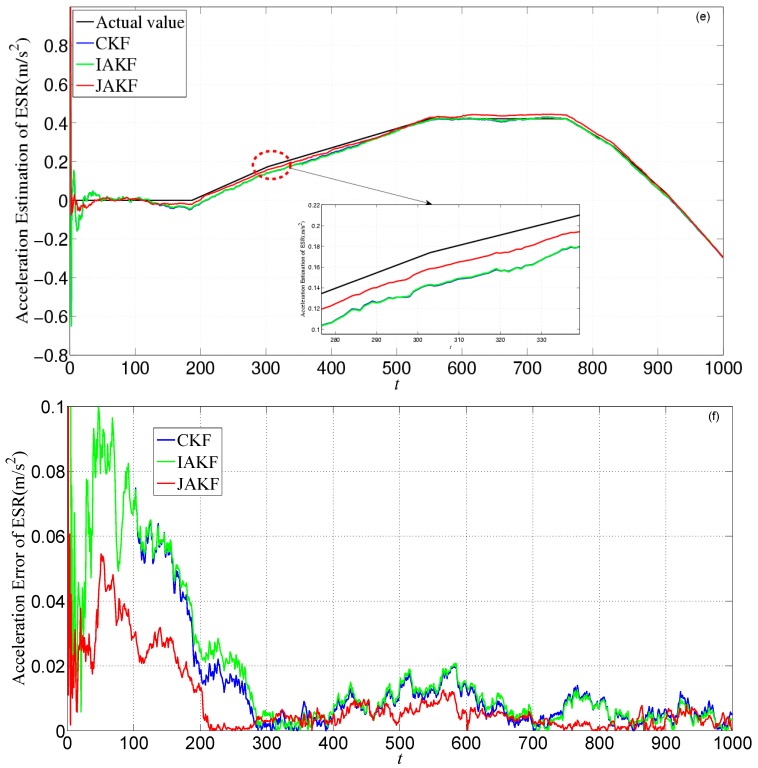
Estimation and error comparison of ESR in the third experiment: (**a**) Displacement estimation of ESR; (**b**) Displacement error of ESR; (**c**) Velocity estimation of ESR; (**d**) Velocity estimation of ESR; (**e**) Acceleration estimation of ESR; (**f**) Acceleration error of ESR.

Figure 19 gives the estimation and error comparison of ESR. After the filters converged, all the measured noises of the three filters fluctuate within the theoretical range. The errors in CKF and IAKF are nearly similar while the JAKF still behaves best w.r.t. accuracy. For example, at the 600th time slot, the error of the displacement is about 0.0412 m in JAKF, while 0.0954 m in CKF and 0.0986 m in IAKF; the error of the velocity is about 0.0428 m/s in CKF and 0.0402 m/s in IAKF, while 0.0125 m/s in JAKF; and the error of the acceleration is about 0.0081 m/s^2^ in JAKF, while 0.0092 m/s^2^ in CKF and 0.0095 m/s^2^ in IAKF. Therefore, the accuracy of JAKF is stable in the situation where the forward car is permitted to change lanes.

Table 5 lists the comparisons of CKF, IAKF and JAKF about root-mean-square error, maximum error, and variance of filtered results.

In Table 5, compared with the results of the first two experiments, the advantages of JAKF in accuracy and stability decline slightly, but the JAKF results are still better than the ones of CKF and IAKF. Table 6 summarizes the ratio of accuracy improvement of JAKF in the three experiments.

In a nutshell, the JAKF extends the single adaptive filter through integrating the respective advantages of Lidar and Radar, and uses the noise variance *R* and the information allocation factor *β* to adjust the filtered results of the local and global filters, respectively, and thus can improve the fault tolerance and accuracy.

## 5. Conclusions

The paper proposed a joint adaptive Kalman filter algorithm called JAKF that combines one Lidar and one Radar to accurately estimate the motion state of the moving car ahead, e.g., the relative position, the relative velocity, and the relative acceleration. For adaptation, JAKF adopts the modified innovation sequence to calculate the measurement noise V-C matrix of each local filter. Since the measurement noise V-C matrix can reflect the performance of the current filter, the information allocation factor can be specified to optimize the multi-sensorial fusion. The simulation and experimental results show that JAKF can always maintain the convergence even when suffering from abnormal noise. JAKF significantly improves the fault tolerance and stability of the estimation system, and meanwhile, it enhances the accuracy of the filtered results.

## Figures and Tables

**Table 1:** The relation between *R* and the accuracy of Lidar.

SNR (*dB*)	80	81	82	83	84	85	86	87
*R*	0.0021	0.0016	0.0013	0.0010	0.0008	0.0006	0.0005	0.0004
Accuracy (*m*)	0.0362	0.0318	0.0284	0.0254	0.0226	0.0201	0.0180	0.0164

**Table 2:** Parameters of URG and ESR.

	URG	ESR
Measuring distance	0.1 m~30 m	0.5 m~60 m
Distance accuracy	±0.03 m	±0.25 m
Scanning angle	±120°	±45°
Angular resolution	0.36°	±1°
Scanning time	25 ms/scan	50 ms/scan

**Table 3:** Error comparisons of CKF, IAKF, and JAKF.

	CKF_URG_	IAKF_URG_	CKF_ESR_	IAKF_ESR_	JAKF
RMS Distance Error (m)	0.0880	0.0479	0.0595	0.0565	0.0320
Distance Variance	2.0833 × 10^4^	2.0831 × 10^4^	2.0833 × 10^4^	2.0832 × 10^4^	2.0831 × 10^4^
RMS Velocity Error (m/s)	0.0702	0.0665	0.0676	0.0669	0.0640
Velocity Variance	6.7979	6.7852	6.7722	6.7699	6.7737
RMS Acceleration Error (m/s^2^)	0.0175	0.0155	0.0184	0.0179	0.0140
Acceleration Variance	4.2133 × 10^−4^	2.2840 × 10^−4^	3.1428 × 10^−4^	3.0290 × 10^−4^	1.8763 × 10^−4^

**Table 4:** Error comparisons of CKF, IAKF, and JAKF in second experiment.

	CKF_URG_	IAKF_URG_	CKF_ESR_	IAKF_ESR_	JAKF
RMS Distance Error (m)	0.0912	0.0883	0.1754	0.1296	0.0685
Distance Variance	2.8775 × 10^4^	2.8744 × 10^4^	2.8771 × 10^4^	2.8774 × 10^4^	2.8773 × 10^4^
RMS Velocity Error (m/s)	0.0367	0.0338	0.0527	0.0551	0.0269
Velocity Variance	6.8020	6.7737	6.7849	6.7872	6.7794
RMS Acceleration Error (m/s^2^)	0.0190	0.0133	0.0205	0.0203	0.0135
Acceleration Variance	0.1355	0.1364	0.1351	0.1348	0.1356

**Table 5:** Error comparisons of CKF, IAKF, and JAKF in third experiment.

	CKF_URG_	IAKF_URG_	CKF_ESR_	IAKF_ESR_	JAKF
RMS Distance Error (m)	0.0779	0.0636	0.0681	0.0636	0.0549
Distance Variance	2.8638 × 10^4^	2.8635 × 10^4^	2.8642 × 10^4^	2.8641 × 10^4^	2.8633 × 10^4^
RMS Velocity Error (m/s)	0.0437	0.0431	0.0431	0.0414	0.0385
Velocity Variance	0.6702	0.6688	0.6711	0.6772	0.6619
RMS Acceleration Error (m/s^2^)	0.0415	0.0380	0.0442	0.0443	0.0368
Acceleration Variance	0.0414	0.0410	0.0420	0.0424	0.0409

**Table 6:** Ratio of accuracy improvement of JAKF in the three experiments.

	JAKF Compare	Displacement	Velocity	Acceleration
The first experiment	CKF	54.93%	27.08%	21.96%
	IAKF	28.28%	19.05%	15.74%
The second experiment	CKF	42.91%	37.85%	36.43%
	IAKF	35.03%	35.79%	16%
The third experiment	CKF	25.14%	13.29%	14.02%
	IAKF	13.68%	9.84%	10.04%
